# Dynamics of Regulatory T-Cells during Pregnancy: Effect of HIV Infection and Correlations with Other Immune Parameters

**DOI:** 10.1371/journal.pone.0028172

**Published:** 2011-11-29

**Authors:** Kelly Richardson, Adriana Weinberg

**Affiliations:** 1 Department of Pediatrics, University of Colorado Denver, Aurora, Colorado, United States of America; 2 Department of Medicine, University of Colorado Denver, Aurora, Colorado, United States of America; 3 Department of Pathology, University of Colorado Denver, Aurora, Colorado, United States of America; University of London, St George's, United Kingdom

## Abstract

**Objectives:**

Regulatory T cells (Treg) increase in the context of HIV infection and pregnancy. We studied Treg subpopulations in HIV-infected and uninfected women during pregnancy and their relationship with inflammation, activation and cell-mediated immunity (CMI).

**Design and Methods:**

Blood obtained from 20 HIV-infected and 18 uninfected women during early and late gestation was used to measure Treg and activated T cells (Tact) by flow cytometry; plasma cytokines and inflammatory markers by ELISA and chemoluminescence; and CMI against varicella-zoster virus (VZV) by lymphocyte proliferation.

**Results and Conclusions:**

Compared with uninfected women, HIV-infected participants had higher frequencies of Treg subpopulations in early pregnancy, including CD4+CD25+FoxP3+%, CD8+CD25+FoxP3+%, CD4+TGFβ+% and CD4+IL10+%. In contrast, Treg frequencies were lower during late pregnancy in HIV-infected compared with uninfected women, including CD8+TGFβ+%, CD4+CTLA4+% and CD8+CTLA4+%. VZV-CMI, which was lower in HIV-infected compared with uninfected pregnant women, was inversely correlated with CD4+FoxP3+%, CD8+FoxP3+% and CD8+TGFβ+% in HIV-infected, but not in uninfected pregnant women. β_2-_microglobulin, neopterin, IL1, IL4, IL8, IL10, IFNγ and TNFα plasma concentrations as well as Tact were higher in HIV-infected compared with uninfected women throughout pregnancy. In HIV-infected, but not in uninfected women, inflammatory, Th1, Th2 and regulatory cytokines increased with higher Treg%, suggesting that inflammation and regulation have a common pathophysiologic origin in the context of HIV infection. In HIV-infected and more commonly in uninfected pregnant women, higher Treg% correlated with lower Tact%. We conclude that Treg have different dynamics during pregnancy in HIV-infected and uninfected women. Higher levels of inflammatory cytokines and lower Treg% during late pregnancy in HIV-infected women may contribute to their increased incidence of maternal-fetal morbidity.

## Introduction

There is growing evidence that regulatory T cells (Treg) strongly influence adaptive immune responses. The role of Treg in down-modulating inflammation and consequently preventing morbidity is clearly established in the context of autoimmune disorders and transplantation [1], [2]. Evidence has been accumulating to support a similar down-modulatory effect of Treg in the context of infectious and neoplastic disorders, but with undesired clinical consequences, such as establishing permissive conditions for chronic infections and tumor growth [3], [4], [5]. The behavior of Treg and their role in the context of HIV infection has been debated. Some studies presented evidence that increased proportions of Treg were present in HIV-infected individuals and favored HIV replication [6], [7], [8], [9], [10], [11], [12], which is typically associated with increased inflammation and activation. Other studies suggested that Treg were decreased in HIV-infected individuals, which contributed to the persistent immune activation associated with HIV infection and created conditions for the development of immune reconstitution syndromes [13], [14], [15]. As immune modulating agents progressively enter the therapeutic arena, it is important to establish the role of Treg in the pathogenesis of HIV infection.

T cell activation is a hallmark of HIV pathogenesis [16], [17], [18]. An excess of both CD4+ and CD8+ activated T cells (Tact) are present in the peripheral blood of HIV-infected individuals and directly or indirectly contribute to the loss of CD4+ T cells [7], [19]. Furthermore, in HAART recipients, increased inflammatory activity has been associated with cardiovascular, hepatic and renal disorders [20], [21], [22], [23], [24], which, together with non-AIDS-related malignancies, account for a growing proportion of morbidity and mortality of HIV-infected individuals in the era of HAART [25], [26].

Pregnancy is associated with increased inflammatory biomarkers and with higher Treg proportions [2], [27], [28], [29], [30], [31]. Pregnancy does not appear to accelerate HIV disease progression or increase the risk of death of HIV-infected women [32]. However, pregnancy has been associated with increased transmission and acquisition of HIV [33], which would be consistent with the generalized immunosuppression as well as with increased activation and immune-regulatory activity during pregnancy [2], [34], [35]. Previous studies showed that inflammatory biomarkers and Tact are higher in HIV-infected pregnant women compared with uninfected pregnant women [27], [28], [34]. The level of inflammatory cytokines may affect the outcome of pregnancy by increasing the risk of premature delivery and of other pregnancy-specific complications [36], [37], [38], [39], [40], [41], [42].

The goal of this study was to define the dynamics of Treg in HIV-infected pregnant women by comparison with uninfected women and to determine the relationship of Treg with cell-mediated immunity (CMI), inflammatory factors and T cell activation during pregnancy.

## Methods

### Study Design

This was a prospective study performed at the Children's Human Immunodeficiency Program at The Children's Hospital in Denver and University of Colorado Hospital. Subjects were HIV-infected and uninfected pregnant women who provided written consent to participate in this study approved by the Colorado Multiple Institution Review Board. All women were seropositive for varicella-zoster virus (VZV). Blood was obtained during the 2^nd^ and 3^rd^ trimesters of pregnancy. Laboratory studies included CD4+ and CD8+ cell enumeration, inflammatory biomarkers, Treg, Tact and VZV -specific CMI and serology in all participants and HIV plasma RNA in HIV-infected women.

### Plasma cytokines and inflammatory biomarkers

IL1, IL4, IL6, IL8, IL10, IFNγ and TNFα were measured with the chemoluminescence commercial kit Quansys [Quansys Biosciences; limit of detection (LOD) of 0.87, 1.78, 0.21, 0.22, 0.25 and 0.73 pg/mL respectively). C-Reactive Protein (CRP), β_2-_microglobulin, CTLA-4 and neopterin were measured with the following kits: Quantikine (R & D Systems; LOD = 0.01 ng/mL), β_2_-Microglobulin ELISA Kit (Immundiagnostik AG; LOD = 0.1 mg/L), CTLA-4 ELISA Kit (Invitrogen; LOD = 0.2 ng/mL) and Neopterin ELISA (IBL, Inc.; LOD = 0.7 nmol/L). All assays were performed according to the manufacturers' instructions, in duplicate wells. Results were considered valid if replicates differed by <2-fold and if positive and negative controls were within the ranges specified by each kit.

### CMI Assays

Peripheral blood mononuclear cells (PBMC) from heparinized blood, separated by ficoll/hystopaque density gradient centrifugation (Sigma Chemical Company), were cryopreserved as previously described [43].

*LPA* was performed as previously described [43]. Stimulation medium consisted of RPMI 1640 with glutamine (Gibco), 10% human AB serum (NABI) and 1% antibiotics (Gibco). PBMC (10^5^ cells/well) were added to quadruplicate wells containing VZV cell lysate, mock-infected control lysate and pokeweed mitogen (Sigma) at 10 µg/mL. After six days at 37°C in 5% CO_2_ atmosphere, cells were pulsed with 20 µCi/mL ^3^H-thymidine and their DNA was harvested 6 h later onto filter mats (Perkin Elmer). Radioactivity on the filters was counted on a microplate scintillation counter (Packard). Assays were considered valid if the pokeweed mitogen-stimulated wells had 10-fold higher counts-per-minute (cpm) than the medium control. Results were expressed as median cpm in VZV-stimulated wells minus median cpm in mock-stimulated control wells.

#### Flow cytometry

The T-cell phenotypes were assessed by flow cytometry using freshly thawed PBMC. Cells were stained using monoclonal antibodies against the following molecules: CD3 (BD Biosciences), CD4 (Beckman Coulter), IL-10 (eBioscience), TGFβ (Cedarlane), FoxP3 (eBioscience), CD25 (BD Biosciences), CTLA4 (BD Biosciences),CD38 (BD Biosciences), and HLADR (BD Biosciences). Total T cells and subpopulations were counted on Guava easyCyte 8HT (Millipore) and analyzed with FlowJo (Treestar). Subpopulations were expressed as percentages of the parent CD4+ or CD8+ T cell populations.

**Statistical analysis** was performed using Prism5 and InStat software (GraphPad). Parametric tests were used when the distribution of the data was normal. Nonparametric tests were used when the distribution was not normal even after log transformation. Significance was defined by a p value <0.05.

## Results

### Demographics

The study enrolled 20 HIV-infected and 18 uninfected pregnant women with mean±SD gestational age of 19±7 and 17±6 weeks, respectively (Table 1). There were more black women among the HIV-infected compared with the uninfected participants (6 vs. 0), reflecting the characteristics of the HIV epidemic in the USA. However, this difference did not reach statistical significance (p = 0.06, Chi square). HIV-infected women also tended to be younger than the uninfected women (p = 0.06) and had significantly lower CD4+ and higher CD8+ cell counts (p≤0.01). All HIV-infected women received HAART for ≥24 days before the first study visit, including 4 who were on HAART before pregnancy. The median (range) of the plasma HIV viral load of the HIV-infected participants decreased, but not significantly, between the entry and the late pregnancy visits: 74 (<20; 61,100) and <20 (<20; 4,130) RNA copies/ml, respectively, p = 0.20 (1-tail paired T test).

**Table 1:** Baseline Characteristics of Study Participants.

*Characteristic*		*HIV-infected*	*Uninfected*	*P value*
Numbers		20	18	n.a.
Years of age (mean±S.D.)		26±5	29±6	0.06*
Race				
	White	11	14	0.06^#^
	Black	6	0	
	Hispanic	3	3	
	Asian	0	1	
Weeks gestation (mean±S.D.)		19±7	17±6	0.207*
CD4 cells/µl (mean±S.D.)		480±217	971±220	**<0.0001** *
CD8 cells/µl (mean±S.D.)		774±437	447±214	**0.01** *
Plasma HIV RNA c/ml (median; range)		74; <20–61,100	n.a.	n.a.
Days of HAART (median; range)		41; 24–337	n.a.	n.a.

*Unpaired T test.

# Chi-square.

Bold facing indicates significant differences.

### Treg dynamics during pregnancy in HIV-infected and uninfected women

**Figure 1:**
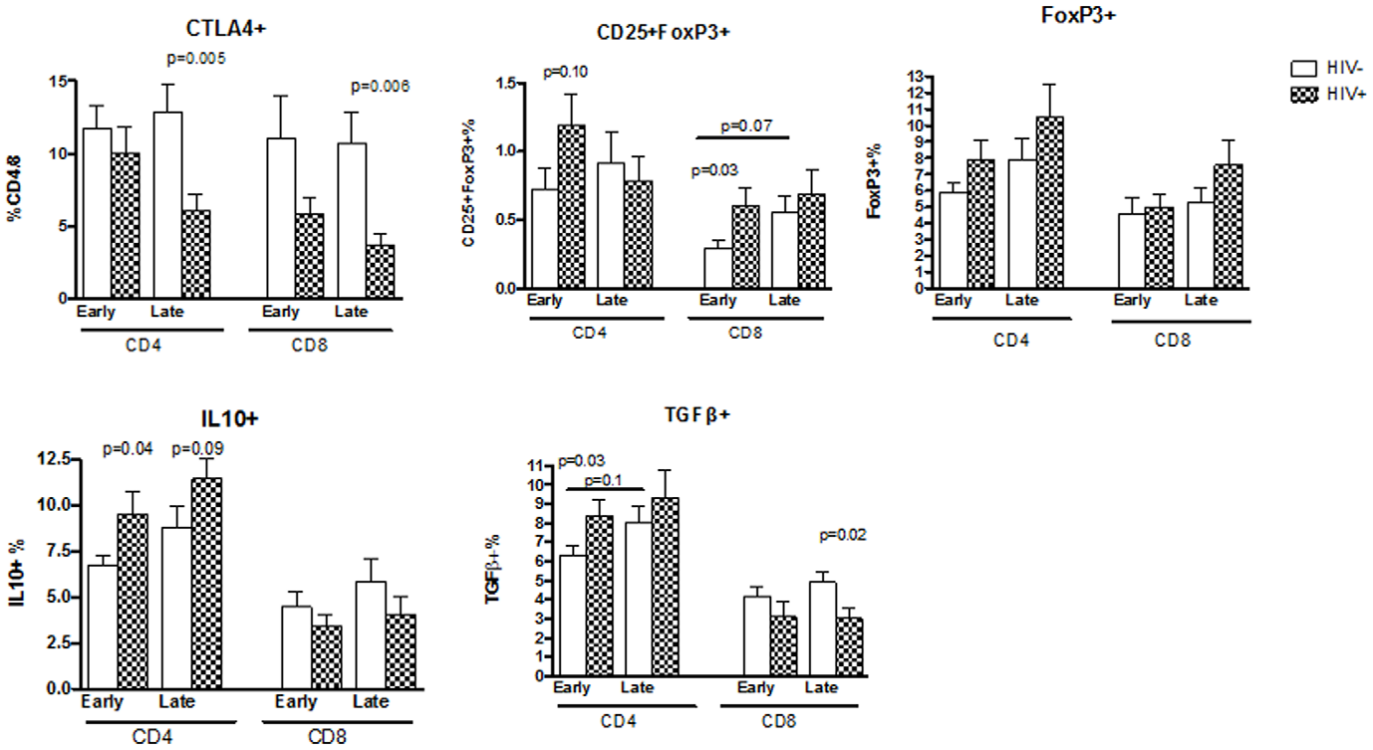
Proportions of Treg in HIV-infected and uninfected women during early and late gestation. Bars represent means and standard error of the mean (SEM) of data generated by flow cytometric analysis of freshly thawed PBMC. The Treg are expressed as a percentage of the parent CD4+ or CD8+ T cell population. The graph titles indicate the Treg subpopulation depicted in each graph. Significant or marginally significant differences between HIV-infected and uninfected women are indicated by p values immediately above each time point (Mann Whitney). Horizontal lines indicate significant or marginally significant changes between early and late pregnancy. Such changes were detected only in uninfected women.

Treg were measured at entry (early pregnancy) and during the 3^rd^ trimester (late pregnancy). The third trimester visit occurred at mean±SEM of 33±6 weeks gestation in both groups of participants. Treg were characterized by the expression of FoxP3, co-expression of FoxP3 and CD25, CTLA4, IL10 and TGFβ (Fig. 1). The last 3 markers were chosen for their ability to provide some information on the Treg mechanisms of action.

In early pregnancy, Treg frequencies were higher or similar in HIV-infected compared with uninfected women. Significant or marginally significant differences were observed for CD4+CD25+FoxP3+% (p = 0.1), CD8+CD25+FoxP3+% (p = 0.03), CD4+TGFβ+% (p = 0.03) and CD4+IL10+% (p = 0.04). In late pregnancy, CD4+IL10+% tended to continue higher in HIV-infected compared with uninfected women (p = 0.09). In contrast, other Treg subpopulations were lower in HIV-infected women, including CD8+TGFβ+% (p = 0.02), CD4+CTLA4+% (p = 0.005) and CD8+CTLA4+% (p = 0.006). In HIV-infected women, there were no significant changes in any of the Treg subpopulations between early and late pregnancy. However, in uninfected women, CD8+CD25+FoxP3+% and CD4+TGFβ+% tended to increase from early to late time points (p of 0.07 and 0.1, respectively).

### Functional CMI during pregnancy and post-partum in HIV-infected and uninfected women

**Figure 2:**
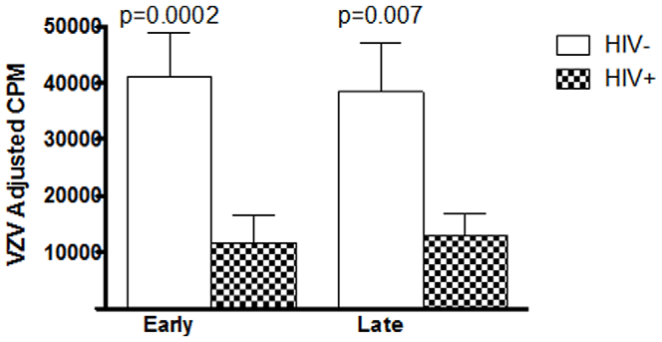
Cell-mediated immunity in HIV-infected and uninfected pregnant women during early and late gestation. Data were generated using VZV- and mock-stimulated cryopreserved PBMC. Bars represent means and SEM of adjusted VZV-specific counts-per-minute (cpm) after subtraction of mock-stimulated cpm. Significant differences between HIV-infected and uninfected women at each time point are indicated by the p values on the graph (unpaired T test using log-transformed data).

VZV-specific CMI was measured in this study. All HIV-infected and uninfected participants in this study were VZV-seropositive. HIV-infected women had significantly lower VZV-CMI compared with those of uninfected women during early and late pregnancy (p of 0.0002 and 0.0007, respectively; Fig. 2). There were no significant changes in VZV-CMI with advancement of pregnancy in either group.

### Plasma cytokines and inflammatory factors in early and late gestation in HIV-infected and uninfected women

**Figure 3:**
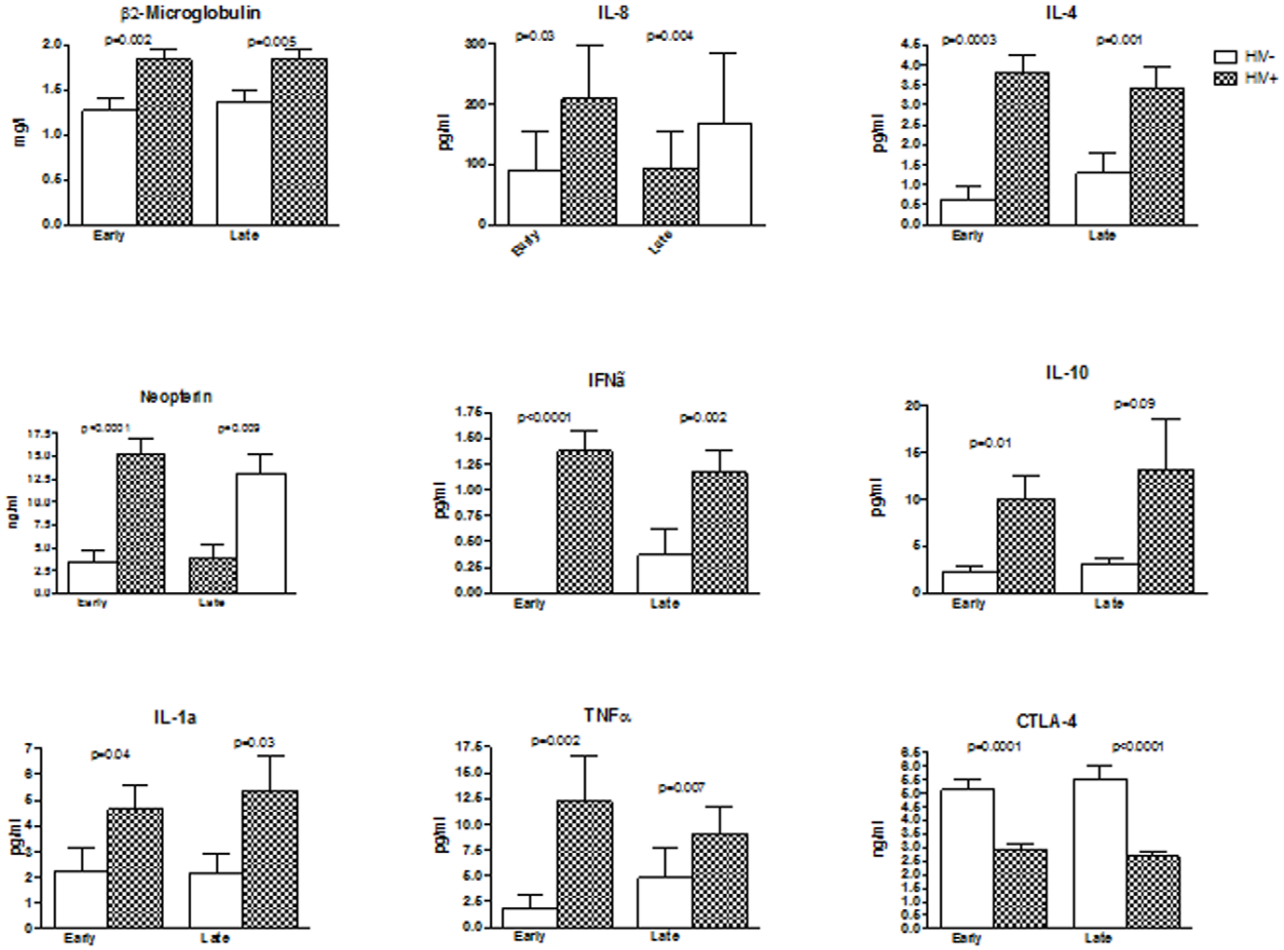
Inflammatory factors and other cytokines in HIV-infected and uninfected women during early and late gestation. Bars represent means and SEM. The analytes are indicated in the graph titles. Significant differences between HIV-infected and uninfected women are indicated by the p values immediately above the bars (Mann Whitney).

We compared the plasma concentrations of CRP, neopterin, β_2_microglobulin, CTLA4, IL1, IL4, IL6, IL8, IL10, IFNγ and TNFα (Fig. 3). All cytokines and inflammatory factors with the exception of CRP, IL6 and soluble CTLA4 showed significantly higher plasma concentrations in HIV-infected compared with uninfected women both in early and late gestation (p<0.0001 to 0.02). In contrast, soluble CTLA4 concentrations were significantly lower in HIV-infected women compared with uninfected controls in early and late pregnancy (p = 0.0001 and <0.0001, respectively). CRP and IL6 levels (data not depicted) were similar in HIV-infected and uninfected women in early (mean ±SEM of 1.6±0.1 vs. 1.0±0.1 ng/ml and 49±26 vs. 26.3±10 pg/ml, respectively) and late pregnancy (1.3±0.1 vs. 1.1±0.1 ng/ml and 64±36 vs. 31±23 pg/ml, respectively). There were no significant changes in plasma cytokines and other biomarkers from early to late pregnancy in either group of subjects.

### T cell activation during and after pregnancy in HIV-infected and uninfected women

**Figure 4:**
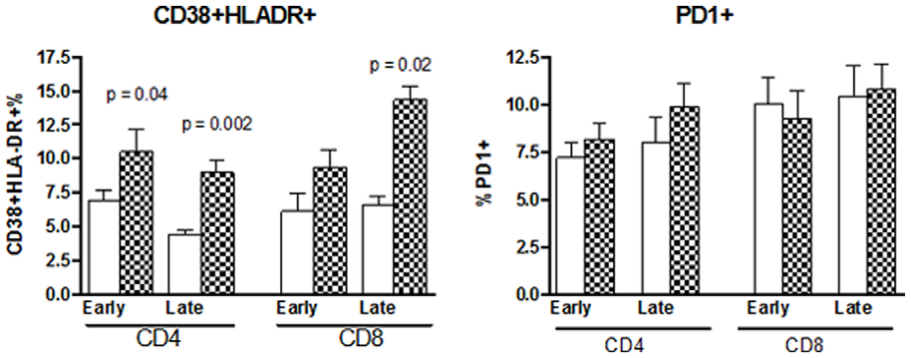
Proportions of Tact in HIV-infected and uninfected women during early and late gestation. Bars indicate mean and SEM proportions of the Tact subpopulations indicated in the title of each graph. Tact are expressed as percentages of the parent CD4+ or CD8+ T cell populations. Significant differences between HIV-infected and uninfected women are indicated by p values immediately above each time point (Mann Whitney test).

Tact were primarily defined by the co-expression of CD38 and HLADR or by cell surface expression of PD-1 on CD4+ or CD8+ T cells. HIV-infected women had significantly higher CD4+CD38+HLADR+% compared with uninfected women in early and late pregnancy (p of 0.04 and 0.002, respectively; Fig. 4) and in CD8+CD38+HLADR+% in late pregnancy (p = 0.002). There were no significant differences in CD8+CD38+HLADR+% in early pregnancy or in PD-1 expression in early or late pregnancy between HIV-infected and uninfected women.

### Relationship of Treg with T cell function, activation and inflammatory factors in HIV-infected and uninfected women in early pregnancy

Correlation analyses were performed for each Treg% subpopulation with CD4+CD38+HLADR+ and CD8+CD38+HLADR+ Tact%; VZV adjusted-cpm; and plasma inflammatory (neopterin and IL8), Th1 (IFNγ), Th2 (IL4) and regulatory (IL10) cytokine concentrations (Table 2). VZV cpm showed significant negative correlations with CD4+FoxP3+%, CD8+FoxP3+% and CD8+TGFβ+% (rho of −0.57 to −0.70; p of 0.004 to 0.03) and a trend towards positive correlation with circulating CD4+CTLA4+% (rho = 0.49, p = 0.07). Participants with higher CD4+CD25+FoxP3+%, CD8+CD25+FoxP3+%, CD4+IL10+%, CD8+IL10+%, CD4+TGFβ+% and CD8+TGFβ+% also had significant or borderline significant higher plasma levels of neopterin, IL8, IFNγ, IL4 or IL10 (rho of 0.48 to 0.60; p of 0.02 to 0.08). There were marginally significant negative correlations of CD4+CD25+FoxP3+% with CD4+CD38+HLADR+% and of CD4+TGFβ+% with CD8+CD38+HLADR+% (rho of −0.58 and −0.54; p of 0.08 and 0.1, respectively).

**Table 2:** Correlation of Treg with Cell-Mediated Immunity, Plasma Inflammatory Factors and Other Cytokines and Tact in HIV-Infected and Uninfected Pregnant Women.

Variable (%)	VZV	Neopt	IL8	IFNγ	IL4	IL10	CD4+	CD8+
	cpm	µg/ml	pg/ml	pg/ml	pg/ml	pg/ml	act	act
**HIV-INFECTED WOMEN**								
CD4+CD25+FoxP3+	-0.19*	0.13	**0.52**	**0.52**	**0.66**	0.32	0.27	***-0.58***
CD8+CD25+FoxP3+	-0.08	***0.45***	***0.50***	**0.51**	0.31	***0.48***	0.14	-0.32
CD4+IL10+	0.29	**0.60**	0.36	***0.48***	***0.50***	***0.48***	0	0.28
CD8+IL10+	-0.05	0.30	***0.48***	***0.48***	0.28	0.19	0.38	0.13
CD4+TGFβ+	-0.17	0.20	0.18	0.08	0.22	**0.60**	***-0.54***	-0.03
CD8+TGFβ+	**-0.65**	-0.03	**0.58**	0.39	0.40	0.36	-0.47	0.09
CD4+FoxP3+	**-0.70**	-0.13	0.32	0.18	0.04	0.09	-0.37	-0.10
CD8+FoxP3+	**-0.57**	-0.12	0.35	0.19	0.06	-0.06	-0.38	-0.09
CD4+CTLA4+	***0.49***	0.21	0.11	0	0.19	0.08	0.05	0.07
CD8+CTLA4+	0.22	0.22	0.23	0	0.27	-0.28	0.08	0.02
**UNINFECTED WOMEN**								
CD4+CD25+FoxP3+	-0.16	0.19	-0.12	0	0.28	0.25	**-0.69**	0.01
CD8+CD25+FoxP3+	-0.12	***-0.54***	0.27	-0.10	0.35	***0.52***	-0.46	0.21
CD4+IL10+	-0.09	0.25	-0.22	0.45	-0.20	-0.14	0.28	0.20
CD8+IL10+	-0.03	0.10	-0.10	0.11	-0.06	0	-0.03	0
CD4+TGFβ+	0.12	0.06	0.31	-0.07	0.16	0.06	-0.35	0.30
CD8+TGFβ+	-0.26	0.28	-0.23	-0.03	0.16	0.4	***-0.58***	0.09
CD4+FoxP3+	0.20	0.11	0.35	-0.45	0.04	0.25	**-0.67**	***-0.58***
CD8+FoxP3+	-0.22	-0.10	0.11	0.08	0.17	0.07	-0.30	-0.09
CD4+CTLA4+	-0.17	**0.59**	0.08	0.24	0	0.21	0.02	-0.37
CD8+CTLA4+	0.15	0.39	-0.08	0.17	-0.19	-0.07	0.14	-0.34

*Numbers represent Spearman correlation coefficients. Bold-facing and underscore indicates significant associations, with p<0.05; bold-facing and italics indicates marginally significant associations with p values of 0.05 to 0.1.

In uninfected pregnant women, Treg% were not associated with CMI (Table 2). Higher CD8+CD25+FoxP3+% was marginally associated with lower concentration of neopterin and higher plasma concentrations of IL10, whereas CD4+CTLA4+% positively correlated with neopterin plasma levels. There were significant or marginally significant negative correlations of CD4+CD25+FoxP3+%, CD8+TGFβ+% and CD4+FoxP3+% Treg with CD4+CD38+HLADR+% and/or CD8+CD38+HLADR+% Tact.

To determine if HIV replication or CD4 depletion were a common link for the associations of Treg with inflammatory factors or with functional CMI described above, we performed correlation analyses of HIV plasma RNA, CD4 cell numbers and CD4% with each Treg subpopulation. These analyses revealed a marginally significant association between CD4+FoxP3+% with plasma HIV RNA (rho = 0.53, p = 0.07).

## Discussion

In early pregnancy, CD4+ and CD8+ Treg subpopulations were generally increased in HIV-infected compared with uninfected women. This included CD4+CD25+FoxP3+, CD8+CD25+FoxP3+, CD4+TGFβ+ and CD4+IL10+ Treg and it was most likely an effect of HIV infection, which is in agreement with previous findings in non-pregnant HIV-infected adults [6], [8], [44], [45]. Our findings, however, contrast with a recently published study of pregnant women, which showed lower CD4+CD25+FoxP3+CD127^low^ Treg frequencies in HIV-infected compared with uninfected women during the 2nd trimester of pregnancy[46]. The duration of HAART of the HIV-infected subjects differed between the two studies, with most of our subjects starting HAART during pregnancy, while in the study by Kolte et al., most subjects were on HAART before pregnancy. Since HAART has been previously shown to decrease the frequency of circulating Treg in HIV-infected individuals [47], the difference in the duration of HAART may explain the difference between the two study results.

More importantly, in late pregnancy several Treg subpopulations, including CD4+CTLA4+%, CD8+CTLA4+% and CD8+TGFβ+% were lower in HIV-infected compared with uninfected pregnant women, and only CD4+IL10+% remained higher in HIV-infected women. Furthermore, no significant increases in Treg% were detected in HIV-infected women between early and late gestation, whereas in uninfected women, we observed a marginally significant increase of CD8+CD25+FoxP3+% and CD4+TGFβ+% over the course of pregnancy. Previous studies also observed Treg increases during normal pregnancy and ascribed them a role in inducing maternal tolerance of the fetus [48], [49], [50], [51], [52]. Blois et al. also determined that CD8+ Treg had a pivotal role as a mediator of the anti-abortogenic effect of progesterone in mice [53]. The lack of Treg% upregulation during late gestation in HIV-infected pregnant women may lead to a premature loss of fetal tolerance and premature delivery. In fact, HIV-infected women have a higher incidence of premature delivery compared with the general population [54], [55], [56]. This was sometimes, but not always, correlated with the use of protease inhibitor-containing HAART [56], [57]. It is conceivable that the use of HAART may exert a downregulatory pressure on Treg subpopulations similar to what was described in non-pregnant HIV-infected adults [47]. Furthermore, we found an association, albeit weak, between plasma HIV load and CD4+FoxP3+% in early pregnancy, which also supports the notion that active HIV replication may increase Treg%, while HAART-mediated control of HIV replication may have the opposite effect. The hypothesis that lack of upregulation of specific Treg subpopulations in late pregnancy is associated with premature delivery needs to be further confirmed in larger studies, because it may lead to new therapeutic interventions.

To gather information on the Treg functionality, we studied the relationship of Treg% with VZV-specific lymphocyte proliferation. We chose VZV CMI as a representative measure because of the high prevalence of VZV infection in the US adult population (>90%) and the well-demonstrated role of VZV CMI in protection against VZV reactivation and herpes zoster [58], [59]. We found that higher CD4+FoxP3+%, CD8+FoxP3+% and CD8+TGFβ+% were significantly associated with lower VZV-specific proliferation in HIV-infected pregnant women. Interestingly, not all Treg subpopulations were associated with decreased CMI in HIV-infected pregnant women, which suggests different specificities of Treg subpopulations. Furthermore, Treg% were not associated with lower CMI in HIV-uninfected pregnant women. It is possible that the association between high Treg% and low CMI in the context of HIV infection is indirect and that both phenomena are linked to active HIV replication. However, it is also conceivable that the higher frequency of Treg in HIV-infected women during early pregnancy allowed us to detect an inhibitory effect on CMI only in this group of participants. This may be due to a higher ratio between Treg and effector memory T cells in HIV-infected compared with uninfected women and/or broader antigen specificity of certain CD4+FoxP3+, CD8+FoxP3+ and CD8+ TGFβ+ Treg in HIV-infected women.

Among the Treg markers used in this study, CTLA4 seemed to behave differently from others. CD4+CTLA4+% and CD8+CTLA4+% were higher in HIV-uninfected compared with infected pregnant women and this difference reached statistical significance in late pregnancy, whereas most other Treg% were higher in HIV-infected women in early pregnancy. Furthermore, soluble CTLA4 plasma concentrations were significantly higher in HIV-uninfected women both in early and late gestation, whereas IL10 plasma concentrations were higher in HIV-infected women. CTLA4 is expressed both by Treg and memory effector T cells and in both circumstances contributes to down-modulation of the cellular immune response. Memory effector T cells quickly upregulate CTLA4 expression upon cognate antigenic stimulation. Binding of CD80 or CD86 ligands to CTLA4 on effector memory T cells decreases their IL2 production and cell cycle progression [60]. In counterpart, CTLA4 expression on Treg allows them to block the activation of naïve T cells. In this study, we did not differentiate between effector memory and Treg expression of CTLA4 [61]. It is conceivable that some of the CTLA4+ T cells measured in this study were effector memory cells expressing CTLA4, which may explain the different dynamics of CTLA4+ T cells compared with other Treg subpopulations. Conversely, CTLA4+ T cells may represent bonafide Treg and their behavior in pregnancy differs from that of other Treg subpopulations. Further studies are needed to elucidate the role of CTLA4 expression in maintenance of pregnancy and the potential role that CTLA4Ig might play as a therapeutic intervention.

We found that HIV-infected women had elevated CD4+CD38+HLADR+% and CD8+CD38+HLADR+% Tact compared with uninfected women, which was in agreement with previous observations in non-pregnant individuals [34]. Insufficient T cell regulation was invoked as a potential contributor to high levels of T cell activation in HIV-infected individuals [13], [15]. In this study, we found negative associations between Treg% and Tact%, which reached statistical significance in uninfected pregnant women and were only marginally significant in HIV-infected participants. This is unlikely to be due to deficient Treg function in the context of HIV infection, because we also observed a strong negative association between Treg% and lymphocyte proliferation in HIV-infected women. A more likely explanation is that in the context of HIV infection Treg and Tact share some of the pathophysiologic mechanisms, partially masking the inhibitory Treg effect on Tact. This would be in agreement with the model proposed by Boasso et al. whereby activation of plasmacytoid dendritic cells by HIV infection would increase both regulation and activation [62].

Plasma concentrations of inflammatory factors, Th1, Th2 and Treg cytokines were consistently higher throughout pregnancy in HIV-infected compared with uninfected pregnant women. High levels of inflammatory factors in non-pregnant HIV-infected adults were previously shown to correlate with increased risk of non-AIDS severe adverse events, such as cardiovascular, hepatic and renal dysfunctions [63]. In pregnancy, high inflammatory factors may lead to obstetric morbid conditions, such as premature delivery, pre-eclampsia and liver disorders [38], [64], [65], [66]. In HIV-infected pregnant women, like in other HIV-infected non-pregnant adults [8], [9], inflammatory factors, Th1, Th2 and Treg cytokines were positively correlated with Treg%, probably reflecting a common pathophysiologic mechanism. However, in uninfected pregnant women, CD8+FoxP3+% were negatively associated with inflammatory markers and positively associated with Treg cytokines signaling that Treg interactions may differ between HIV-infected and uninfected pregnant women. It is important to confirm these findings and further characterize Treg subpopulations and inflammatory factors that play a direct role in the outcome of pregnancies, which may provide new avenues for therapeutic interventions.
